# Variation in post-colonoscopy colorectal cancer across colonoscopy providers in English National Health Service: population based cohort study

**DOI:** 10.1136/bmj.l6090

**Published:** 2019-11-13

**Authors:** Nicholas E Burr, Edmund Derbyshire, John Taylor, Simon Whalley, Venkataraman Subramanian, Paul J Finan, Matthew D Rutter, Roland Valori, Eva J A Morris

**Affiliations:** 1 Cancer Epidemiology Group, Institute of Cancer and Pathology and Institute of Data Analytics, University of Leeds, Leeds LS2 9JT, UK; 2 Mid Yorkshire Hospitals NHS Trust, Pinderfields General Hospital, Wakefield, UK; 3 Royal Liverpool and Broadgreen University Hospitals NHS Trust, Liverpool, UK; 4 Leeds Teaching Hospitals NHS Trust, St James’s University Hospital, Leeds, UK; 5 John Goligher Colorectal Unit, St James’s University Hospital, Leeds, UK; 6 University Hospital of North Tees, Hardwick, Stockton on Tees, UK; 7 Northern Institute for Cancer Research, Newcastle University, Newcastle, UK; 8 Gloucestershire Hospitals NHS Foundation Trust, Gloucester, UK

## Abstract

**Objectives:**

To quantify post-colonoscopy colorectal cancer (PCCRC) rates in England by using recent World Endoscopy Organisation guidelines, compare incidence among colonoscopy providers, and explore associated factors that could benefit from quality improvement initiatives.

**Design:**

Population based cohort study.

**Setting:**

National Health Service in England between 2005 and 2013.

**Population:**

All people undergoing colonoscopy and subsequently diagnosed as having colorectal cancer up to three years after their investigation (PCCRC-3yr).

**Main outcome measures:**

National trends in incidence of PCCRC (within 6-36 months of colonoscopy), univariable and multivariable analyses to explore factors associated with occurrence, and funnel plots to measure variation among providers.

**Results:**

The overall unadjusted PCCRC-3yr rate was 7.4% (9317/126 152), which decreased from 9.0% in 2005 to 6.5% in 2013 (P<0.01). Rates were lower for colonoscopies performed under the NHS bowel cancer screening programme (593/16 640, 3.6%), while they were higher for those conducted by non-NHS providers (187/2009, 9.3%). Rates were higher in women, in older age groups, and in people with inflammatory bowel disease or diverticular disease, in those with higher comorbidity scores, and in people with previous cancers. Substantial variation in rates among colonoscopy providers remained after adjustment for case mix.

**Conclusions:**

Wide variation exists in PCCRC-3yr rates across NHS colonoscopy providers in England. The lowest incidence was seen in colonoscopies performed under the NHS bowel cancer screening programme. Quality improvement initiatives are needed to address this variation in rates and prevent colorectal cancer by enabling earlier diagnosis, removing premalignant polyps, and therefore improving outcomes.

## Introduction

Colorectal cancer is a major public health problem in the United Kingdom. Each year, more than 40 000 new patients are diagnosed as having the disease and there are around 16 000 deaths.^1^ International comparisons have revealed that the survival rate of patients with colorectal cancer in the UK lags behind that attained by many of our economic neighbours. This finding is largely because of a high mortality rate soon after diagnosis, particularly among older people. The best survival rates are observed in patients who are diagnosed at an early stage. Therefore, outcomes can be improved by optimising the diagnostic process.

Colonoscopy is the main test for diagnosing colorectal cancer. This procedure also has the potential to prevent the disease by removing precancerous lesions, and so it is an important tool to help improve outcomes.^2^ Unfortunately, the test is not 100% accurate and cancers can appear within months or years after a colonoscopy that is negative for cancer. The World Endoscopy Organisation defines these cases as post-colonoscopy colorectal cancers (PCCRCs).^3^ Evidence suggests that up to 700 patients are diagnosed as having PCCRCs each year in the English National Health Service (NHS).^4^ Therefore, reducing the incidence of these cancers is vital.

Colonoscopy services in the NHS are under pressure. Currently, over 650 000 colonoscopies are undertaken each year, but demand is growing substantially and there is concern that the workforce needed to meet this demand is insufficient. Although major efforts have been made to ensure the NHS delivers high quality colonoscopy services, for example, the Joint Advisory Group on Gastrointestinal Endoscopy (JAG) accreditation scheme, not all providers have been mandated to participate. As a result, there is the potential for variation in the quality of colonoscopy services across the country; unless this variation is quantified, it cannot be addressed.

The British Society of Gastroenterology has proposed that PCCRC rates should be used as a benchmark for colonoscopy quality.^5^ Recently, the World Endoscopy Organisation^6^ provided methods to calculate rates so that comparisons can be made among institutions and jurisdictions. Our study aimed to apply these methods to compare PCCRC rates within three years of colonoscopy (PCCRC-3yr) among all providers, and to quantify variation in colonoscopy quality across the English NHS.

## Methods

### Study population

We obtained study data from the UK Colorectal Cancer Intelligence Hub’s colorectal cancer data repository (CORECT-R), in which routine cancer datasets are linked to provide a rich resource of population based data.^7^ Our study population consisted of people who had undergone a colonoscopy in the English NHS between 1 January 2005 and 31 December 2013 and who subsequently developed colorectal cancer (International Classification of Diseases, 10th revision (ICD10) codes C18-20). We used linked inpatient and outpatient Hospital Episode Statistics (HES) data (OPCS4 codes H20-22), English NHS bowel cancer screening programme (BCSP), and National Cancer Registration and Analysis Service datasets available within the resource. The BCSP offers screening (through an initial guaiac based faecal occult blood test to determine eligibility for colonoscopy; soon to be replaced by a faecal immunochemical test) every two years to women and men aged 60-74. After age 74, people can request additional screening tests.^8^

The colonoscopy data included all procedures performed by NHS providers, private patients treated at NHS centres, and procedures performed by independent providers paid for by the NHS. We excluded tumours that involve the appendix (ICD10 C181) or those with a neuroendocrine, lymphoma, squamous, or melanoma morphology. Only adenocarcinoma subtypes were included because they are the main focus for detecting and preventing cancer after removal of serrated and adenomatous polyps. We limited our cohort to those with colorectal cancer that was diagnosed within three years of colonoscopy and with data extracted for analysis at colonoscopy level. The cohort was then split into three groups for analysis based on the year of colonoscopy: 2005-07, 2008-10, and 2011-13.

### PCCRC-3yr rate calculation

Methods produced by the World Endoscopy Organisation were used to calculate the PCCRC-3yr rate.^6^ We categorised colonoscopies into positive and negative tests according to whether a cancer was diagnosed within six months of the test. When a colorectal cancer occurred within the first six months after a colonoscopy, this was a true positive test; when a colorectal cancer appeared between six and 36 months after a colonoscopy, this was a false negative test. We defined cancers as detected cancers if they were preceded by a true positive colonoscopy and as a PCCRC-3yr if they were preceded by a false negative colonoscopy. This 6-36 month window for defining a PCCRC-3yr rate is advocated by the World Endoscopy Organisation so that institutions and jurisdictions can be compared by using the same metric. The definition does not require the index test to be negative for adenomas.

We appreciate that the terms false negative and true positive normally depend on a gold standard, which does not exist for the diagnosis of colorectal cancer and its precursors. Therefore, we assume that the gold standard is cancer diagnosed within three years of a colonoscopy. Some cancers that appear after a negative colonoscopy might be new, rapidly growing cancers or cancers arising from premalignant polyps. Thus, in this instance we use the term gold standard to refer to the potential of colonoscopy to detect or prevent cancers that will present within three years after the colonoscopy.

Some people in the cohort underwent multiple colonoscopies. For these people, we only included true positive and false negative colonoscopies that were recorded closest to the time of diagnosis, which is in accordance with World Endoscopy Organisation methods.

### Cohort characteristics

We identified colonoscopies undertaken within the BCSP, but the indication for the procedure was otherwise unknown. However, other patient level data were available and so we investigated relevant factors that might increase the risk of PCCRC. These data included age at diagnosis and colonoscopy stratified into age bands (≤60, 61-70, 71-80, and >80). We used these cut-off points because at the time of the study bowel cancer screening invitations began at age 60. Other variables were sex, socioeconomic status (based on the income domain of the index of multiple deprivation 2007),^9^ and a comorbidity score based on diagnostic HES codes in the year before cancer diagnosis (subdivided into 0, 1, 2, and ≥3). The comorbidity score was the Charlson score,^10^ with the exception of malignancy because all patients had a diagnosis of colorectal cancer. We included several cancer related variables: stage, classified as stage I-IV, or unknown when data were missing; and colonic location of the tumour defined as caecum, right sided (ascending colon to transverse colon; C18.0-18.4), left sided (splenic flexure to rectosigmoid junction; C18.5-18.7 and C19.0), rectal (C20), or unspecified when no location was given (C18.8-18.9). We also recorded when cancers were diagnosed through an “emergency presentation” because this method of referral is associated with worse outcomes for colorectal cancer in the UK.^11^

Additionally, we identified people with a previous HES coded diagnosis of inflammatory bowel disease, Crohn’s disease, ulcerative colitis (ICD10 codes K50-51), or diverticular disease (ICD10 code K57). We also identified people with a previous diagnosis of colorectal cancer or previous colonoscopy, which was any colonoscopy performed before the test that diagnosed the colorectal cancer or the false negative test when the cancer was not diagnosed by colonoscopy.

### Statistical analyses

We calculated the proportion of people with cancer who had true positive and false negative colonoscopies, and the PCCRC-3yr rate for each three year group according to World Endoscopy Organisation methods. We identified the healthcare provider that performed each colonoscopy by using the unique five digit codes recorded in HES data and from the screening centre in the BCSP. NHS organisations change over time (for example, hospital mergers), therefore historical organisations were mapped to current providers, as they existed on 1 January 2018. Some providers had low workloads, which made comparison of PCCRC-3yr rates with other providers potentially unreliable.^12^ Therefore, we performed an a priori power calculation to determine the minimum number of cases that might be needed to detect an important difference among providers. We used a PCCRC-3yr rate of 7.5% for this calculation based on a previous rate of 8.6% from a study that covered an earlier time period^4^ and an assumption that this rate would have reduced. A doubling of this rate to 15% was deemed to be unacceptably high. Based on this number and 80% power at the 0.05 significance level, a colonoscopy provider would need to detect 96 cancers in any given time period for there to be sufficient statistical power to be labelled as a significant outlier.

We calculated the PCCRC-3yr rate for each year in the study period, and used χ^2^ for trend as a significance test. Additional analyses were undertaken for subgroups: patients with inflammatory bowel disease; people who had colonoscopies as part of the BCSP; and a “non-surveillance” group that excluded people who had colonoscopies within the BCSP and those with a diagnosis of inflammatory bowel disease. We estimated the number of people with PCCRC if the unadjusted rate for each year in the study period was reduced to the 75th centile as a potential benchmark for a minimum standard, and then at the rate achieved by the BCSP as an aspirational target. This would indicate the potential number of cancers that could be diagnosed earlier or prevented if the overall rate is improved to these levels.

We explored the change in PCCRC rate over time for each provider by grouping each unit in the earliest cohort (2005-07) into fifths based on the PCCRC-3yr rate. Similar fifths were then produced for the 2011-13 period and the change in fifths was compared for each colonoscopy provider.

We built multilevel, logistic regression models to determine factors associated with the occurrence of PCCRC-3yr. The models were structured to reflect that people could have undergone multiple colonoscopies, with the person fitted as a random effect. The dependent variable was the occurrence of PCCRC-3yr. Exploratory variables were year of colonoscopy, age group at colonoscopy, sex, index of multiple deprivation income category (fifths), comorbidity score, and a previous HES coded diagnosis of inflammatory bowel disease or diverticular disease, previous colorectal cancer, and previous colonoscopy; additionally, whether the colonoscopy was performed within the BCSP, independent sector, or a non-BCSP service.

We produced unadjusted and risk adjusted funnel plots by using these models and the Spiegelhalter method^13^ to quantify variation in the rate of PCCRC-3yr for each colonoscopy provider in each time period. Funnel plots are a useful graphical display for institutional variation; they are constructed as a scatterplot with superimposed control limits, which represent two and three standard deviations from the mean. The smaller the sample size, the wider the control limits.^14^ Providers who fall outside the control limits have PCCRC rates that are statistically significantly different from the national rate, independently of the case mix factors adjusted for and colonoscopy workload, and so indicate unwarranted variation. We also created unadjusted and risk adjusted histograms to show the PCCRC-3yr rate for each colonoscopy provider in each of the three time periods. Non-modifiable risk factors were used for these models: year of colonoscopy, age group at colonoscopy, sex, index of multiple deprivation income category (fifths), comorbidity score, previous HES coded diagnosis of inflammatory bowel disease or diverticular disease, previous colorectal cancer, and previous colonoscopy; additionally whether the colonoscopy was undertaken within the BCSP or independent sector. All analyses were performed using Stata version 15 (StataCorp, College Station, TX).

### Patient and public involvement

A patient and public group was involved at the design stage of the study. Results have been reported back to the group. The group will be involved in preparing dissemination materials which will be fed back to the colonoscopy providers and released through the national charities Bowel Cancer UK and Cancer Research UK.

## Results

**Fig 1:**
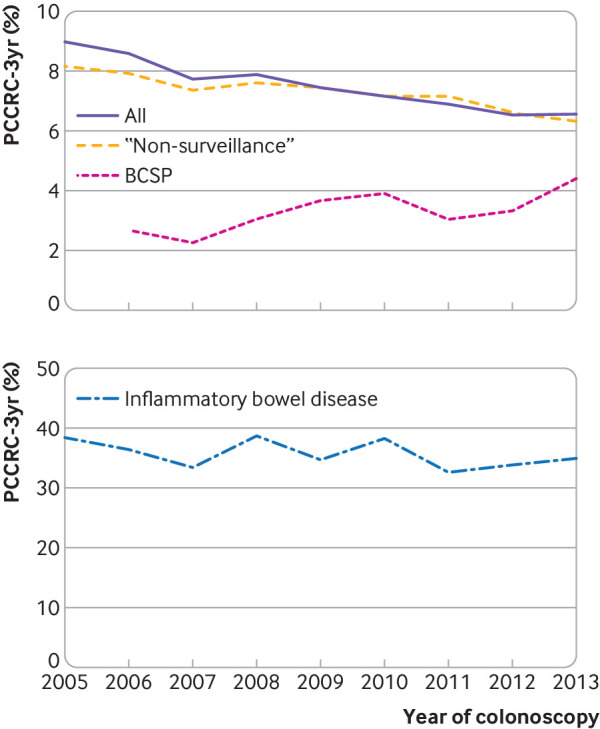
Trends in unadjusted rates of post-colonoscopy colorectal cancer within three years of investigation (PCCRC-3yr) for each year of colonoscopy. Top panel: all colonoscopies, “non-surveillance,” and UK bowel cancer screening programme (BCSP). Bottom panel: colonoscopies in people with an admission code for inflammatory bowel disease. “Non-surveillance” colonoscopies are those in people not diagnosed as having inflammatory bowel disease and those not performed within the BCSP

From an estimated population in England of over 55 million in 2016,^15^ our study included 126 152 colonoscopies in 121 402 people who were diagnosed as having colorectal cancer within three years of their investigation. In this population, 9317 (7.4%) were classified as PCCRC-3yr. There was a statistically significant reduction in the PCCRC-3yr rate from 9.0% for colonoscopies performed in 2005 to 6.5% for those performed in 2013 (P<0.01) (fig 1). The PCCRC-3yr rate in people with inflammatory bowel disease was 38.3% for colonoscopies performed in 2005 and 35.5% for those performed in 2013 (P=0.24). For the BCSP colonoscopies, the rate increased from 2.7% in 2005 to 3.6% in 2013, but did not reach statistical significance (P=0.06). For colonoscopies performed in people with non-inflammatory bowel disease and those that were not conducted within the BCSP, there was a significant reduction in the rate from 8.2% in 2005 to 7.3% in 2013 (P<0.01). Most providers stayed within the same fifth of the PCCRC-3yr rate in the 2005-07 and 2011-13 groups (supplementary table S3).

Table 1 shows the proportion of detected cancers and the PCCRC-3yr rate in relation to the characteristics of the population and the colonoscopies they underwent. The PCCRC-3yr rate was higher after colonoscopies performed in the earlier study periods; in women; among people with a higher comorbidity score; in those with a previous HES code for inflammatory bowel disease or diverticular disease; in people with previous colorectal cancer; and with increasing numbers of previous colonoscopy tests. The PCCRC-3yr rate was higher in the oldest age group than in the youngest age group. Additionally, the rate was much lower in those who had colonoscopies as part of the BCSP (PCCRC-3yr rate 3.6%) than in those who had colonoscopies performed by independent providers outside of the NHS (9.3% across the entire study period). The PCCRC-3yr rate was higher in people with early (stage I), late (stage IV), and unknown stage cancers, and in those with cancer in the proximal colon. Significantly more people were diagnosed as having PCCRC-3yr after an emergency presentation, which is associated with poorer outcomes.

**Table 1:** Detected cancers and PCCRC-3yr rate in relation to characteristics of study population and type of colonoscopy. Values are No (%) unless stated otherwise

Characteristic	Colonoscopies with colorectal cancer diagnosed within three years			PCCRC-3yr rate (%)
	Total	True positive	False negative	
**Total**	126 152	116 835	9317	7.4
**Year of colonoscopy**				
2005-07	31 336	28 711 (25)	2625 (28)	8.4
2008-10	44 165	40 856 (35)	3309 (36)	7.5
2011-13	50 651	47 268 (40)	3383 (36)	6.7
**Age at colonoscopy**				
≤60	22 185	20 731 (18)	1454 (16)	6.6
61-70	38 521	36 153 (31)	2368 (25)	6.1
71-80	42 833	39 479 (34)	3354 (36)	7.8
>80	22 613	20 472 (18)	2141 (23)	9.5
**Sex**				
Male	73 459	68 344 (58)	5115 (55)	7.0
Female	52 693	48 491 (42)	4202 (45)	8.0
**IMD income category**				
Most affluent	27 180	25 169 (22)	2011 (22)	7.4
2	28 331	26 246 (22)	2085 (22)	7.4
3	26 479	24 533 (21)	1946 (21)	7.3
4	23 857	22 078 (19)	1779 (19)	7.5
Least affluent	20 305	18 809 (16)	1496 (16)	7.4
**Charlson comorbidity score**				
0	93 684	87 738 (75)	5946 (64)	6.3
1	21 576	19 590 (17)	1986 (21)	9.2
2	6438	5715 (5)	723 (8)	11.2
≥3	4454	3792 (3)	662 (7)	14.9
**Inflammatory bowel disease**				
No	123 428	108 893 (98)	8348 (90)	6.8
Yes	2724	1756 (2)	968 (10)	35.5
**Diverticular disease**				
No	94 437	88 813 (76)	5624 (60)	6.0
Yes	31 715	28 022 (24)	3693 (40)	11.6
**Previous colorectal cancer**				
No	124 513	115 708 (99)	8805 (95)	7.1
Yes	1639	1127 (1)	512 (5)	31.2
**Previous colonoscopy**				
No	105 972	100 541 (86)	5431 (58)	5.1
Yes	20 180	16 294 (14)	3886 (42)	19.3
**Colonoscopy within BCSP**				
No	109 512	100 788 (86)	8724 (94)	8.0
Yes	16 640	16 047 (14)	593 (6)	3.6
**Colonoscopy by independent provider**				
No	124 143	115 013 (98)	9130 (98)	7.4
Yes	2009	1822 (2)	187 (2)	9.3
**Cancer characteristics**				
Stage:				
I	21 474	19 597 (17)	1877 (20)	8.7
II	34 605	32 613 (28)	1992 (21)	5.8
III	33 240	31 186 (27)	2054 (22)	6.2
IV	13 564	12 252 (10)	1312 (14)	9.7
Unknown	23 269	21 187 (18)	2082 (22)	8.9
Colonic location:				
Rectum	31 492	29 496 (25)	1996 (21)	6.3
Distal colon	40 783	38 450 (33)	2333 (25)	5.7
Proximal colon	27 383	25 143 (22)	2240 (24)	8.2
Caecum	20 978	19 057 (16)	1921 (21)	9.2
Colon NOS	5516	4689 (4)	827 (9)	15.0

BCSP=English NHS bowel cancer screening programme; IMD=index of multiple deprivation; NOS=not otherwise specified; PCCRC-3yr=post-colonoscopy colorectal cancer within three years of colonoscopy.

After multivariable analysis for the factors which can be assessed at the time of colonoscopy, which excludes stage and colonic location, these associations were sustained and all remained statistically significant (P<0.01) (table 2).

**Table 2:** Odds of developing PCCRC-3yr in relation to characteristics of study population and type of colonoscopy

Characteristic	Unadjusted			Adjusted	
	Odds ratio (95% CI)	P value		Odds ratio (95% CI)	P value
**Year of colonoscopy**					
2005-07	1	—		1	—
2008-10	0.89 (0.84 to 0.93)	<0.01		0.86 (0.82 to 0.91)	<0.01
2011-13	0.78 (0.74 to 0.83)	<0.01		0.70 (0.66 to 0.74)	<0.01
**Age at colonoscopy**					
≤60	1	—		1	—
61-70	0.93 (0.87 to 1.00)	0.05		1.02 (0.94 to 1.09)	0.67
71-80	1.21 (1.14 to 1.29)	<0.01		1.02 (0.96 to 1.10)	0.50
>80	1.49 (1.39 to 1.60)	<0.01		1.17 (1.09 to 1.27)	<0.01
**Sex**					
Male	1	—		1	—
Female	1.16 (1.11 to 1.21)	<0.01		1.15 (1.10 to 1.20)	<0.01
**IMD income category**					
Most affluent	1	—		1	—
2	0.99 (0.93 to 1.06)	0.86		0.98 (0.91 to 1.04)	0.50
3	0.99 (0.93 to 1.06)	0.83		0.98 (0.91 to 1.05)	0.52
4	1.01 (0.94 to (1.08)	0.97		0.97 (0.91 to 1.04)	0.44
Least affluent	1.00 (0.93 to 1.07)	0.9		0.93 (0.86 to 1.00)	0.27
**Charlson comorbidity score**					
0	1	—		1	—
1	1.50 (1.42 to 1.58)	<0.01		1.36 (1.28 to 1.44)	<0.01
2	1.87 (1.72 to 2.03)	<0.01		1.62 (1.48 to 1.76)	<0.01
≥3	2.58 (2.36 to 2.81)	<0.01		2.17 (1.98 to 2.38)	<0.01
**Inflammatory bowel disease**					
No	1	—		1	—
Yes	7.60 (7.00 to 8.24)	<0.01		4.93 (4.50 to 5.40)	<0.01
**Diverticular disease**					
No	1	—		1	—
Yes	2.08 (1.99 to 2.17)	<0.01		1.88 (1.79 to 1.97)	<0.01
**Colonoscopy within BCSP**					
No	1	—		1	—
Yes	0.4 (0.39 to 0.46)	<0.01		0.68 (0.62 to 0.74)	<0.01
**Colonoscopy by independent provider**					
No	1	—		1	—
Yes	1.29 (1.11 to 1.51)	<0.01		1.63 (1.39 to 1.91)	<0.01
**Previous colorectal cancer**					
No	1	—		1	—
Yes	5.95 (5.35 to 6.62)	<0.01		2.24 (2.00 to 2.52)	<0.01
**Previous colonoscopy**					
No	1	—		1	—
Yes	4.41 (4.22 to 4.62)	<0.01		3.29 (3.13 to 3.46)	<0.01

### **BCSP=English NHS bowel cancer screening programme; IMD=index of multiple deprivation; PCCRC-3yr=post-colonoscopy colorectal cancer within three years of colonoscopy.**

### Institutional comparison

**Fig 2:**
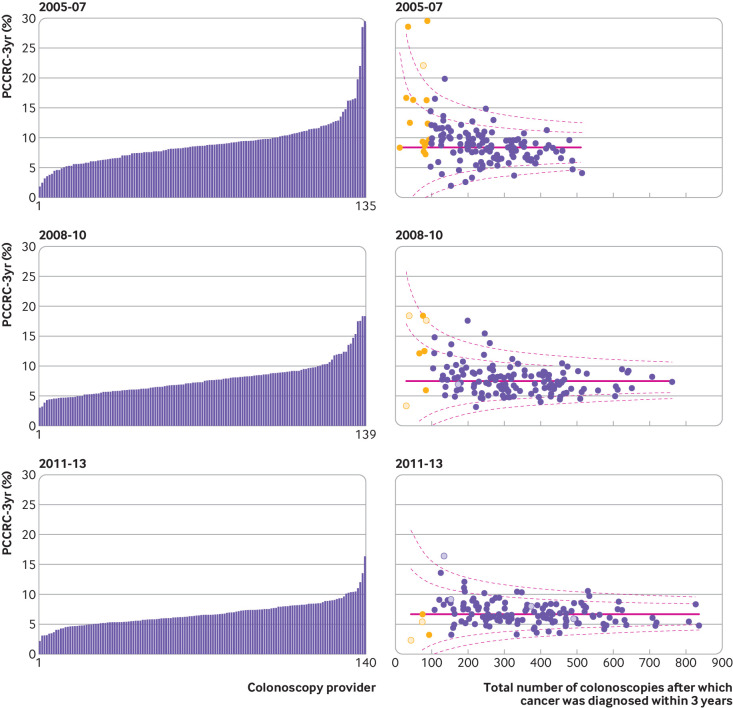
Unadjusted variation in rates of post-colonoscopy colorectal cancer within three years of investigation (PCCRC-3yr) by provider for 2005-07, 2008-10, and 2011-13. In funnel plots each dot represents an individual colonoscopy provider. Dashed lines represent 95% and 99.8% control limits outside national PCCRC-3yr rate (solid line). X axis is number of detected cancers plus PCCRC-3yr cancers diagnosed in the period. Yellow dots indicate providers who diagnosed less than 96 cancers in the given period. Hollow dots represent independent colonoscopy providers

**Fig 3:**
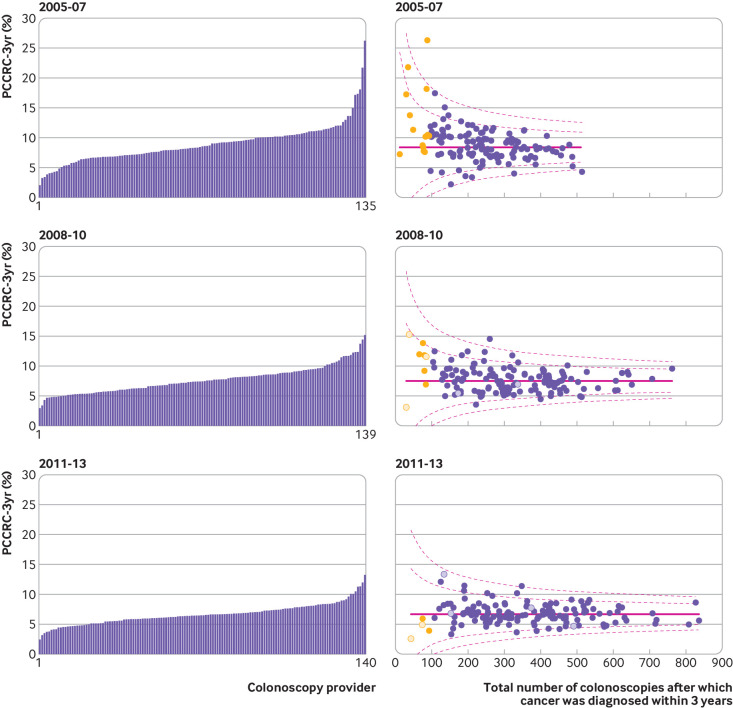
Adjusted variation in rates of post-colonoscopy colorectal cancer within three years of investigation (PCCRC-3yr) by provider for 2005-07, 2008-10, and 2011-13. In funnel plots each dot represents an individual colonoscopy provider. Dashed lines represent 95% and 99.8% control limits outside national PCCRC-3yr rate (solid line). X axis is number of detected cancers plus PCCRC-3yr cancers diagnosed in the period. Adjusted for non-modifiable risk factors: year of colonoscopy, age group at colonoscopy, sex, index of multiple deprivation income category (fifths), comorbidity score, previous Hospital Episode Statistics coded diagnosis of inflammatory bowel disease or diverticular disease, previous colorectal cancer, previous colonoscopy, and whether the colonoscopy was within English NHS bowel cancer screening programme or by independent provider. Yellow dots indicate providers who diagnosed less than 96 cancers in the given period. Hollow dots represent independent colonoscopy providers

A total of 135, 139, and 140 colonoscopy providers were operating in the English NHS during the time periods 2005-07, 2008-10, and 2011-13, respectively. Figure 2 and figure 3 show unadjusted and adjusted institutional comparisons for each of the time periods (see also table 3).

**Table 3:** Institutional variation in risk adjusted and unadjusted PCCRC-3yr rate for each time period. Values are numbers unless stated otherwise

PCCRC-3yr rate	2005-07	2008-10	2011-13
**Adjusted**			
Highest PCCRC-3yr rate (%)*	16.3	14.4	12.8
Lowest PCCRC-3yr rate (%)*	3.4	3.5	3.2
Colonoscopy providers	135	139	140
Above 99.8% CL	1	1	1
Above 95% CL	7	8	10
Within 95% CL	120	125	124
Below 95% CL	6	5	5
Below 99.8% CL	1	0	0
**Unadjusted**			
Highest PCCRC-3yr rate (%)*	19.9	17.6	16.4
Lowest PCCRC-3yr rate (%)*	2.0	3.2	3.2
Colonoscopy providers	135	139	140
Above 99.8% CL	5	5	2
Above 95% CL	13	12	11
Within 95% CL	111	116	120
Below 95% CL	4	6	7
Below 99.8% CL	2	0	0

CL=control limit; PCCRC-3yr=post-colonoscopy colorectal cancer within three years of colonoscopy.

* Providers that detected at least 96 cancers.

Across each time period we found significant variation in unadjusted and adjusted PCCRC-3yr rates. In 2011-13, unadjusted rates ranged from 3.2% to 16.4%, and adjusted rates ranged from 3.2% to 12.8%. Supplementary table 1 shows the centiles for the range of unadjusted PCCRC-3yr rates in 2011-13; the best performing 5% had an unadjusted PCCRC-3yr rate of 4% and the worst performing 5% had a rate of 10.4%.

In the unadjusted funnel plot for the years 2011-13, two providers were outside the upper 99.8% control limit, with 11 providers outside the upper 95% control limit, which indicates higher PCCRC-3yr rates than expected. In contrast, seven providers had rates below the lower 95% control limit, which suggests they had lower PCCRC-3yr rates than expected. No providers were below the lower 99.8% control limit. After risk adjustment for the non-modifiable factors identified above, one provider was above the upper 99.8% control limit, with 10 providers outside the upper 95% control limit. In contrast, there were five providers with rates below the lower 95% control limit. No providers had rates below the 99.8% control limit. Figure 2, figure 3, and table 3 show the results for the years 2005-07, 2008-10, and 2011-13.

We estimated the potential benefit of higher quality colonoscopy by calculating the reduction in PCCRC-3yr if a 6.7% rate, which was found in the 2011-13 cohort, was reduced across the study period. Supplementary figure S1 shows the results of these estimates. Overall, if we reduced the rate to 5.5% or 3.6% as achieved in the BCSP, for the entire study period there would potentially be 168 or 435 fewer patients with PCCRC each year, respectively.

## Discussion

### Principal findings

Our study uses World Endoscopy Organisation methods and robust population based data across the entire English NHS. Although PCCRC-3yr rates are declining, in the most recent cohort the rate was 6.5%. We found a much lower PCCRC-3yr rate for colonoscopies performed as part of the BCSP (3.6%). If this aspirational rate had been achieved for the entire study period, more than 3900 patients with colorectal cancer could have been diagnosed earlier or received preventative treatment.

Although PCCRC-3yr rates fell during each successive time period, we still observed major discrepancies among providers, which persisted after adjustment for associated risk factors. This variation needs to be minimised to increase early diagnosis rates and improve colorectal cancer outcomes. PCCRC-3yr rates for colonoscopies undertaken by independent providers were higher than those reported for NHS providers. These independent providers are increasingly being used to meet the rising demand for colonoscopies.

We found a lower incidence of PCCRC-3yr for colonoscopies performed as part of the BCSP. All BCSP colonoscopies are performed within JAG accredited screening centres by colonoscopists who have undergone an additional accreditation process.^16^ BCSP colonoscopies are of a high standard, with high adenoma detection and caecal intubation rates.^16^ Screening colonoscopists adhere to strict performance criteria, including an unadjusted caecal intubation rate greater than 92% and an adenoma detection rate greater than 40%. Adenoma detection rate correlates with PCCRC, therefore it is reasonable to assume that the high quality of colonoscopies in the BCSP contributes to the reduced PCCRC-3yr rates reported in this study.^17^ This important finding shows that when standards are applied rigorously, quality can improve. Another reason for the decrease in PCCRC-3yr could be because of improvements in endoscopic equipment, including adjuncts to improve detection. A recent randomised study showed increased adenoma number detection with newer generation endoscopes.^18^

In contrast to the BCSP, we found a higher PCCRC rate after colonoscopies undertaken in the independent sector. In recent years there has been a marked increase in demand for colonoscopies in the UK and NHS services have not been able to deliver the number of investigations required, principally because of workforce constraints. Therefore, the independent sector has been used to relieve the backlog of NHS waiting list procedures and we anticipate that they will have generally undertaken “low risk” colonoscopies for which the chance of finding cancer is reduced. We suggest that people at higher risk of developing PCCRC (for example, those with inflammatory bowel disease, genetic conditions predisposing them to colorectal cancer, or large polyps) will have remained under NHS care and so the higher rates of PCCRC in the independent sector might be a cause for concern; however, more detailed data are required to fully quantify the risks. Additional research into provider characteristics that influence colonoscopy outcomes would provide valuable information on how to optimise NHS colonoscopy services. For example, do outcomes differ among providers who do and do not deliver JAG^19^ authorised colonoscopy training? While we were not able to perform these analyses in our study, further studies using new data sources, such as the new National Endoscopy Database and the Private Healthcare Information Network, are proposed to deliver this additional information.

We found that it is important to determine risk factors and define high risk groups so that endoscopists are more aware of the potential for PCCRC. Our data support established risk factors, including increasing age at colonoscopy,^20^ female sex,^20^ ^21^ more comorbidities,^22^ diverticular disease,^22^ ^23^ and inflammatory bowel disease.^24^ Previous colorectal cancer and multiple previous colonoscopies also had strong associations with PCCRC rates. Some of these factors might be associated with incomplete colonoscopy, which is a strong predictor of PCCRC risk.^20^ In this event, it is important that a timely repeat procedure or alternative test is performed, such as cross sectional imaging if clinically appropriate.

The PCCRC-3yr rate for people with inflammatory bowel disease was over three times that of the entire study cohort, an association that has been reported in previous studies.^24^ ^25^ Despite a significant improvement in rates in people without inflammatory bowel disease, the PCCRC-3yr rate in people with inflammatory bowel disease remained constant throughout our study period (P=0.24). Societal guidelines now advocate surveillance on an annual basis for some people with inflammatory bowel disease who are at high risk. While these UK guidelines were published in 2010,^26^ they do not seem to have impacted on the PCCRC-3yr rate (although the data are relatively immature, with colonoscopy data from only three years after their introduction). Additional work to monitor guideline implementation and impact on PCCRC rates is warranted, not least because colorectal cancer is a serious complication of inflammatory bowel disease, and surveillance colonoscopy is a considerable burden for patients and healthcare providers. In the context of surveillance, detection of some patients with PCCRC could reflect the success of the procedure in identifying colorectal cancer early. Therefore, with more colonoscopies being performed, an increase in PCCRC rates will be inevitable, although it is unlikely that this accounts entirely for the increased PCCRC rates found in our study. Despite guidelines for surveillance,^26^ unfortunately these are poorly adhered to.^27^ Quality measures will need to be addressed, which should be the focus of future studies.

### Strengths and limitations

Previous studies have shown the rates of PCCRC are highly sensitive to the method used for calculation and the quality of the data to which that method is applied.^4^ It is vital to have consistent methods to enable reliable benchmarking and comparison of rates. Such an approach would identify what might be possible, but also would highlight areas of underperformance that could benefit from quality improvement interventions. Our study used standardised methods from the World Endoscopy Organisation to calculate PCCRC-3yr rates from a population based dataset that includes all cancers diagnosed in England and all colonoscopies associated with cancer undertaken as part of the screening programme or to investigate symptoms. It is not feasible to perform these analyses on single centre or local datasets because there is often a time lag of several years between the negative test and subsequent pathology. Furthermore, people might be diagnosed as having cancer in a different centre to where the colonoscopy was performed, and therefore, not be captured using local data. National datasets circumvent this problem to a large extent.

The study is potentially limited because the data are from routinely collected clinical codes, and therefore are subject to ascertainment bias. We believe this risk to be low because previous validation studies have shown 96% accuracy for routine data collected in HES and over 99% accuracy in the National Cancer Registry and Analysis Service.^28^ ^29^ The dataset is taken from an NHS administrative dataset that is used for reimbursement and so we expect the numbers of procedures not included in this dataset to be extremely low; however, this possibility cannot be discounted entirely. In addition, there will be a small number of colonoscopies undertaken outside of the NHS by independent providers.

Because we based the study on administrative data, there were important potential causal factors that we could not consider in these analyses. For example, details on whether lesions were incompletely resected,^30^ the presence of large or multiple adenomas, the extent of bowel preparation, failure to book or non-compliance with recommendations for repeat shorter interval testing are all relevant. Unfortunately, such data are not routinely collected in English population datasets. Service level factors exist at each institution that could account for some of the variation seen. Additionally, some institutions perform more procedures with a higher risk of colorectal cancer and therefore PCCRC, such as inflammatory bowel disease surveillance and surveillance after the endoscopic removal of large polyps or early cancers. These variables should be considered in any future research into the cause of PCCRC.

### Conclusions and policy implications

Overall, there has been a sustained improvement in the PCCRC-3yr rate in England. This probably reflects the national initiatives put in place to improve the quality of colonoscopy in England since 2003, overseen initially by the National Endoscopy Team,^31^ and subsequently by the JAG and the British Society of Gastroenterology. For example, most endoscopy services in England are now participating in JAG accreditation, which involves achieving certain standards for service and training, including monitoring performance of colonoscopy and implementing interventions to address poor performance. Additionally, there has been a national colonoscopy training programme since 2003.^31^ There is some evidence that these interventions have improved colonoscopy performance,^32^ ^33^ ^34^ particularly completion of the procedure and appropriate adenoma detection rates, but further work is required to confirm their impact. It is also noteworthy that providers outside the NHS, which have only recently engaged in JAG accreditation,^35^ had a higher overall rate of PCCRC-3yr than NHS providers. It will be important to determine if gaining JAG accreditation helps to close the gap in PCCRC rates between NHS and non-NHS providers. A recent study from Canada, where no such quality improvement initiatives have been mandated, found no reduction in the PCCRC-3yr rate in those aged 50-74 between 1996 and 2010.^36^

Benchmarks need to be set for minimum acceptable standards and aspirational targets. These benchmarks have not been defined for PCCRC-3yr rates. The 25% centile from the range of unadjusted PCCRC-3yr rates was 5.5% and the BCSP rate was 3.6%; these levels would be reasonable benchmarks for a minimum standard and aspirational target, respectively. Reduced rates could be achievable because colorectal cancer detected under the BCSP are smaller lesions, and therefore more likely to be missed.^37^ Furthermore, rates were falling up to the end of 2013; if this trend has continued, recent rates are already likely to be lower.

We need to define causal factors for PCCRC to target people most at risk and implement quality improvement interventions. A large study of PCCRC risk factors found that in a cohort of over 1000 patients with PCCRC, 46% had at least one factor that might have been avoidable.^20^ In the UK, a recent cohort study by Anderson and colleagues found that approximately 70% of PCCRCs might be because of avoidable factors, such as missed or incompletely resected lesions at previous colonoscopy.^38^ These results suggest that despite the improvements over time observed in our study, further reduction in PCCRC-3yr rates is possible.

Serious potential implications exist after a failed test, not limited to colorectal cancer investigations. After a false negative test patients and healthcare professionals might ignore significant symptoms, and delay or avoid repeat investigation. False negative tests also represent a missed opportunity to prevent a future cancer, and the patient might have been spared the consequences of the diagnosis. Potential costs are incurred for patients and healthcare funders and providers of a false negative test: a second colonoscopy or other test might be required; treatment might have to be given for cancer that could have been unnecessary; and cancers are probably more advanced and might have metastasised. Finally, there is the psychological impact of a delayed diagnosis; even if the prognosis is unaffected, patients and their relatives will probably believe their outlook is worse because of the delay.

There is robust evidence that endoscopic screening prevents colorectal cancer,^39^ and the removal of adenomatous polyps reduces death from colorectal cancer.^40^ However, by improving the quality of colonoscopy there is the potential for over diagnosis^41^ because polyps will be detected that prompt surveillance in some people who will not die from colorectal cancer. Reassuringly, recent studies have shown a low risk of colorectal cancer in those with small polyps found at their index test.^42^ ^43^ As a result of this evidence, surveillance guidelines might change to try and reduce the number of unnecessary colonoscopies.

In conclusion, we suggest that the PCCRC-3yr rate is a key performance indicator of the quality of colonoscopy. A minimum standard of up to 5.5% and an aspirational target of up to 3.6% could be applied as quality standards. Substantial unwarranted variation in PCCRC-3yr rates exists among colonoscopy providers in England. PCCRC is largely avoidable and targeted measures are required to reduce rates for all colonoscopy providers, improve earlier detection, and reduce mortality rates from this preventable disease.

**What is already known about this topic**

Post-colonoscopy colorectal cancer (PCCRC) is a key indicator for the quality of a colonoscopy service, but information on its incidence has not been routinely available
Previous studies have used different approaches that make benchmarking and comparison of rates among providers unreliable
The World Endoscopy Organisation has recently produced standardised methods to calculate rates so that comparisons can be made among institutions and jurisdictions

**What this study adds**

This study used World Endoscopy Organisation methods and found wide variation in PCCRC rates within three years of investigation among colonoscopy providers in England
Rates declined from 9.0% for colonoscopies performed in 2005 to 6.5% for those performed in 2013 (P<0.01)
The English NHS bowel cancer screening programme rates were much lower (3.6%) than those found for independent colonoscopy providers (9.3%)
By reducing the national PCCRC-3yr rate to an aspirational target of up to 3.6%, many cancers could be prevented or diagnosed at an earlier stage

## Web extra

Extra material supplied by authors

**Web appendix:** Supplementary materials

**Web appendix:** Visual summary

## Notes

Contributors: NEB and ED are joint first authors. MDR, RV, and EJAM are joint senior authors. EJAM, RV, MDR, NEB, and ED conceived and designed the study. SW, NEB, and EM carried out the data linkage and preparation of the extracts of data from the CORECT-R within the National Colorectal Cancer Intelligence Hub. NEB, JT, and EM undertook the statistical analysis. All authors assisted in the interpretation of the data, drafting the manuscript, and approved the final version. EM, RV, and MDR contributed equally and are joint senior authors. NEB, EM, RV, and MDR are the guarantors. The corresponding author attests that all listed authors meet authorship criteria and that no others meeting the criteria have been omitted.

Ethical approval: The CORECT-R resource, and analyses based upon the data within it, has received approval from the South West-Central Bristol research ethics committee (18/SW/0134).

Data sharing: This project involves data derived from patient level information collected by the NHS, as part of the care and support of patients with cancer.

The lead author (NEB) affirms that the manuscript is an honest, accurate, and transparent account of the study being reported; that no important aspects of the study have been omitted; and that any discrepancies from the study as planned (and, if relevant, registered) have been explained.
