# Supplementary material for: Variation in post-colonoscopy colorectal cancer across colonoscopy providers in English National Health Service: population based cohort study
Source: BMJ. 2019 Nov 13;367:l6090. doi: 10.1136/bmj.l6090 (PMC6849511; doi:10.1136/bmj.l6090)
Supplement: Supplementary file 1 — Web appendix: Supplementary materials [file burn051410.ww.pdf]

Supplementary materials

Figure S1: Number of PCCRC-3yr observed, and expected if the rate were reduced to 5.5% or 3.6% as achieved in the UK Bowel Cancer Screening Programme.

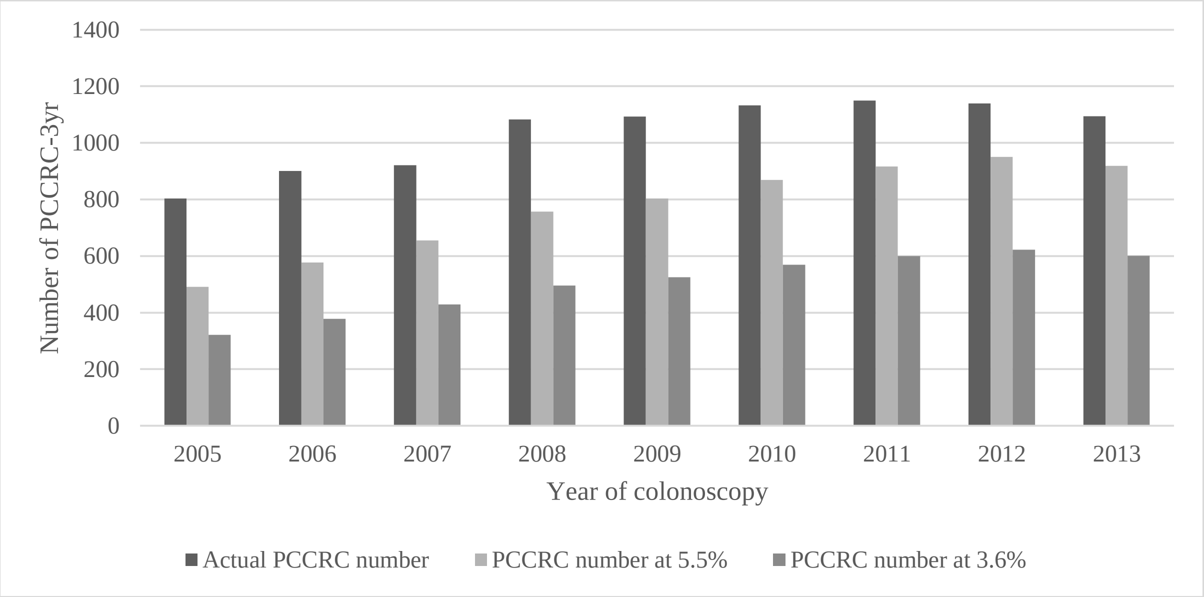

Table S1: Percentiles showing the spread of the unadjusted PCCRC-3yr rate for each 3-year period.

	PCCRC-3yr rate (%)		
**Percentile**	**2005 to 2007**	**2008 to 2010**	**2011 to 2013**
1%	2.6	3.3	3.2
5%	4.6	4.7	4.0
10%	5.4	4.9	4.8
25%	6.8	6.0	5.5
50%	8.8	7.4	6.7
75%	10.5	9.1	8.1
90%	12.7	11.9	9.1
95%	16.3	13.8	10.4
99%	28.6	18.4	13.6

Table S3. Change in the PCCRC-3yr rate for each provider from the earliest cohort (2005 to 2007) to the latest (2011 to 2013).

	Adjusted movement 2005 to 2013		Unadjusted movement 2005 to 2013	
	**Number of providers**	**%**	**Number of providers**	**%**
No change	25	19	67	50
No change or movement of one quintile higher or lower	75	56	120	89
1 quintile lower PCCRC-3yr rate	26	19	32	24
2 quintiles lower PCCRC-3yr rate	17	13	6	4
3 quintiles lower PCCRC-3yr rate	6	4	1	1
4 quintiles lower PCCRC-3yr rate	6	4	0	0
1 quintile higher PCCRC-3yr rate	24	18	21	16
2 quintiles higher PCCRC-3yr rate	18	13	6	4
3 quintiles higher PCCRC-3yr rate	9	7	2	1
4 quintiles higher PCCRC-3yr rate	4	3	0	0
