# Supplementary material for: Variation in post-colonoscopy colorectal cancer across colonoscopy providers in English National Health Service: population based cohort study
Source: BMJ. 2019 Nov 13;367:l6090. doi: 10.1136/bmj.l6090 (PMC6849511; doi:10.1136/bmj.l6090)
Supplement: Supplementary file 2 — Web appendix: Visual summary [file burn051410.vs.pdf]

thebmj Visual summary

I'd like to see your colon again in...

Implications for frequency of colorectal cancer surveillance from two studies

Colonoscopy and biopsy are the cornerstone of the diagnosis of colorectal cancer. For those with negative test results, different countries have different policies about whether and how often to reapeat such tests. Two studies published simultaneously in *The BMJ* investigate how often neoplasms are identified after negative tests at different time points, and by different providers. This graphic summarises their results.

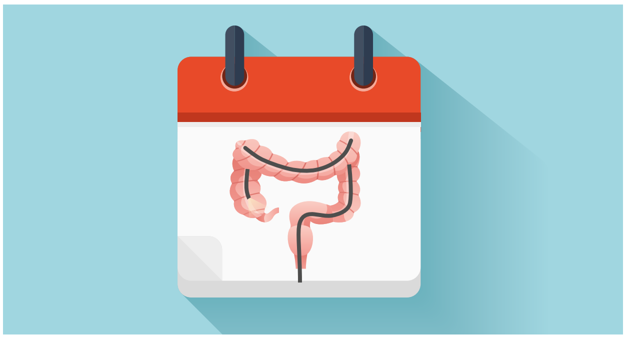

Study 1: Follow-up of negative colonoscopy

Heisser and colleagues find that detection of advanced neoplasms within 10 years of a colonoscopy is rare, suggesting that current US and German guidelines of 10 year colonoscopy screening intervals may be adequate. However, potential sex differences suggested by some studies require further study.

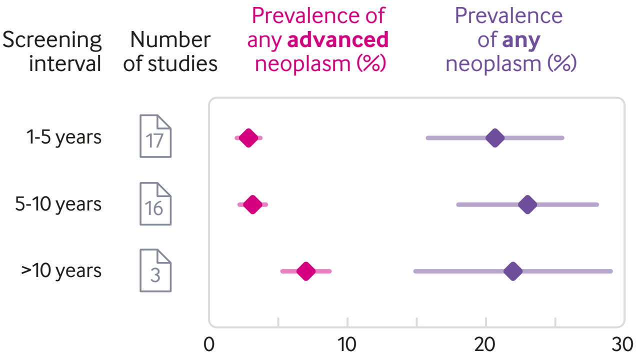

Study characteristics

	Systematic review and meta-analysis		28 studies included
	Patients with average risk of colorectal cancer and a negative previous colonoscopy result	40 to 68	Mean age at follow-up colonoscopy

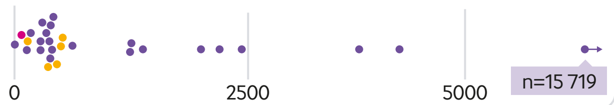

Study 2: Quality of colonoscopy provider

Burr and colleagues investigated the occurrence of colorectal cancer within three years of a colonoscopy where no cancer was found. This is a key measure of the quality of colonoscopy and represents a missed opportunity to diagnose cancer earlier or even prevent it. Rates have fallen, but are still too high, and there is wide variation among colonoscopy providers in England.

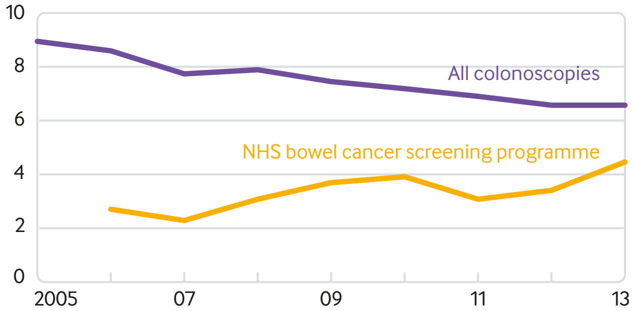

Study characteristics

Cohort study	126 152 colonoscopies in 121 402 individuals
All individuals undergoing colonoscopy in the English NHS between 2005 and 2013, and subsequently diagnosed as having a colorectal cancer up to three years after investigation	

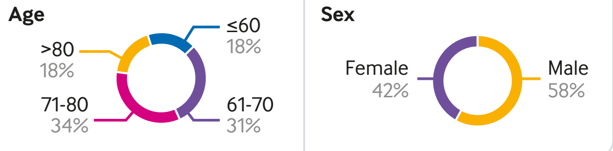

They also found that rates of post-colonoscopy colorectal cancer were higher for colonoscopies undertaken by independent providers than for those performed by NHS providers. Some of the lowest rates are in the NHS bowel cancer screening programme, where the quality of colonoscopy is high.

Overall findings

With high quality colonoscopy, screening more frequently than every 10 years appears to offer little benefit. However, a shorter interval may be required after lower quality screening. It also seems that individual factors such as age and sex should be taken into acount to determinine how often to screen. Further research is required to set screening intervals on a more individual basis.

© 2019 BMJ Publishing Group Ltd.

Outcomes at follow-up of negative colonoscopy in average risk population: systematic review and meta-analysis

Heisser et al

thebmj Read the full article online http://bit.ly/BMJcolfol

Variation in post-colonoscopy colorectal cancer across colonoscopy providers in English National Health Service: population based cohort study

Burr et al

thebmj Read the full article online http://bit.ly/BMJvarcol

See more visual summaries http://www.bmj.com/infographics

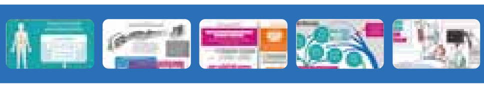

Disclaimer: This infographic is not a validated clinical decision aid. This information is provided without any representations, conditions, or warranties that it is accurate or up to date. BMJ and its licensors assume no responsibility for any aspect of treatment administered with the aid of this information. Any reliance placed on this information is strictly at the user's own risk. For the full disclaimer wording see BMJ's terms and conditions: http://www.bmj.com/company/legal-information/
